# Leucine Modulates Mitochondrial Biogenesis and SIRT1-AMPK Signaling in C2C12 Myotubes

**DOI:** 10.1155/2014/239750

**Published:** 2014-10-07

**Authors:** Chunzi Liang, Benjamin J. Curry, Patricia L. Brown, Michael B. Zemel

**Affiliations:** ^1^Department of Nutrition, University of Tennessee, Knoxville, 1215 W. Cumberland Avenue, 229 Jessie Harris Building, Knoxville, TN 37996-1920, USA; ^2^Ension, Inc., 11020 Solway School Road, Suite 108, Knoxville, TN 37931, USA; ^3^NuSirt Biopharma, 11020 Solway School Road, Suite 109, Knoxville, TN 37931, USA

## Abstract

Previous studies from this laboratory demonstrate that dietary leucine protects against high fat diet-induced mitochondrial impairments and stimulates mitochondrial biogenesis and energy partitioning from adipocytes to muscle cells through SIRT1-mediated mechanisms. Moreover, *β*-hydroxy-*β*-methyl butyrate (HMB), a metabolite of leucine, has been reported to activate AMPK synergistically with resveratrol in C2C12 myotubes. Therefore, we hypothesize that leucine-induced activation of SIRT1 and AMPK is the central event that links the upregulated mitochondrial biogenesis and fatty acid oxidation in skeletal muscle. Thus, C2C12 myotubes were treated with leucine (0.5 mM), alanine (0.5 mM), valine (0.5 mM), EX527 (SIRT1 inhibitor, 25 *μ*M), and Compound C (AMPK inhibitor, 25 *μ*M) alone or in combination to determine the roles of AMPK and SIRT1 in leucine-modulation of energy metabolism. Leucine significantly increased mitochondrial content, mitochondrial biogenesis-related genes expression, fatty acid oxidation, SIRT1 activity and gene expression, and AMPK phosphorylation in C2C12 myotubes compared to the controls, while EX527 and Compound C markedly attenuated these effects. Furthermore, leucine treatment for 24 hours resulted in time-dependent increases in cellular NAD^+^, SIRT1 activity, and p-AMPK level, with SIRT1 activation preceding that of AMPK, indicating that leucine activation of SIRT1, rather than AMPK, is the primary event.

## 1. Introduction

Impaired mitochondrial function in skeletal muscle is one of the major predisposing factors to metabolic diseases, such as insulin resistance, type 2 diabetes, and cardiovascular diseases [1]. Indeed, lower mitochondrial content and decreased expression of oxidative enzymes are observed in patients with type 2 diabetes [2]. SIRT1 and AMP-activated protein kinase (AMPK) are known to promote mitochondrial biogenesis and oxidative capacity and prevent the mitochondrial dysfunction in skeletal muscle [3, 4].

SIRT1, a nicotinamide adenine dinucleotide- (NAD^+^-) dependent deacetylase, is the key enzyme that mediates caloric restriction- (CR-) induced longevity in mammals [5]. By sensing intracellular NAD^+^/NADH ratio, SIRT1 regulates target gene expression via changing acetylation status of histones of transcriptional factors, such as peroxisome proliferator-activated receptor gamma coactivator 1-alpha (PGC-1*α*), tumor suppressor p53 (p53), nuclear factor kappa-light-chain-enhancer of activated B cells (NF-*κ*B), and forkhead box O3 (FOXO3) [6], resulting in modulation of wide range of cellular fundamental processes, including DNA repairing, energy metabolism, and cell apoptosis [7, 8]. Overexpression and activation of SIRT1 protect against high fat diet- (HFD-) induced metabolic abnormalities in mice, such as insulin resistance, glucose intolerance, and liver steatosis, without extending their lifespan [9, 10]. Therefore, small molecules that could activate SIRT1 and mimic the CR impacts have drawn considerable attention.

Leucine, a branched-chain amino acid (BCAA), plays a distinct role in energy metabolism in addition to its pivotal function in protein synthesis [11, 12]. For example, leucine promotes energy partitioning from adipocytes to muscle cells, leading to decreased lipid storage in adipocytes and increased fat utilization in muscle cells [13]. Leucine administration increases insulin sensitivity and glucose tolerance by promoting glucose uptake and fatty acid oxidation in skeletal muscle in HFD-fed mice [14–17]. In fact these effects are mediated partially through SIRT1-dependent pathway, as* Sirt1* knockout significantly attenuates these effects [18, 19]. Further, recent data indicate that leucine can directly activate SIRT1 by promoting the enzyme affinity for its substrates and NAD^+^ [18], resulting in elevated mitochondrial biogenesis and fatty acid oxidation in both adipocytes and myotubes [20, 21].

HMB, a minor metabolite of leucine, has been reported to stimulate AMPK phosphorylation synergistically with metformin, resulting in significant increases in insulin sensitivity and glucose tolerance in mice [22]. Similar to SIRT1, AMPK is an evolutionary conserved enzyme and acts as an energy status sensor via intracellular AMP or AMP/ATP ratio in eukaryotes [3]. In response to nutrient restriction, activated AMPK promotes a cell catabolic shift with increased ATP production to rescue the cellular fuel crisis [23]. Furthermore, phosphorylated AMPK is highly associated with SIRT1 activation in both* in vivo* and* in vitro* studies [5, 24], and part of these two enzymes signaling pathways are overlapped [25].

These findings provide a mechanistic framework for leucine-modulation of mitochondrial biogenesis [21, 26]. We hypothesize that leucine activation of SIRT1 and AMPK is the major event that regulates fatty acid oxidation and mitochondrial biogenesis in skeletal muscle. Accordingly, we examined the effects of leucine, valine (branched-chain amino acid control), and alanine (nonbranched chain amino acid control) on mitochondrial content, mitochondrial biogenesis-related gene expression, SIRT1 activity, and AMPK phosphorylation in C2C12 myotubes. In addition, we used EX-527 (SIRT1 selective inhibitor) and Compound C (specific AMPK inhibitor) to probe the roles of each enzyme in leucine-modulation of energy metabolism in C2C12 myotubes.

## 2. Materials and Methods

### 2.1. Cell Culture

C2C12 myoblast cells were seeded at a density of 1.2 × 10^6^ cells per well in 6-well plates and incubated in Dulbecco's modified eagle medium (DMEM) containing 4.5 g/L D-glucose, 10% fetal bovine serum (FBS), and 1% penicillin-streptomycin at 37°C and 5% CO_2_. After the cells reach 90% confluence, the medium was switched to a standard differentiation medium (DMEM supplemented with 2% horse serum and 1% penicillin-streptomycin) for 2 to 4 days. The differentiation medium was changed every other day to allow 90% of the cells to fully form myotubes (3–5 days later) before additional treatments began.

The dosages of reagents were 0.5 mM for leucine, 0.5 mM for alanine, 0.5 mM for valine, 100 nM for resveratrol, 25 *μ*M for EX527, 25 *μ*M for Compound C, and 50 *μ*M for AICAR. The incubation lengths were from 1 to 48 hours as indicated in the figure legend.

### 2.2. RNA Extraction and Quantitative Real-Time PCR (RT-PCR) Analyses

Total RNA was extracted from C2C12 myotubes using Ambion Totally RNA Isolation Kit (Ambion, Inc., Austin, TX, USA) according to the manufacturers' instructions. The RNA content was determined using NanoDrop ND-1000 Spectrophotometer (NanoDrop Technologies Inc., Wilmington, DE, USA). RNA quality was assessed by the 260 nm/280 nm ratio (1.8–2.0) and 260 nm/230 nm ratio (2.0). The mRNA expression of selected genes related to mitochondrial biogenesis, including* Sirt1*,* Sirt3*,* PGC-1α*, cytochrome c oxidase subunit 5b (*Cox5b*), heat shock cognate protein 1 (*Hspd 1*), and* Cox2,* was analyzed using a TaqMan Universal PCR Master Mix kit (Applied Biosystem) according to the manufacturers' instructions. The primers and probes sets were obtained from Applied Biosystems TaqMan Gene Expression Assays primers and probe set collection. The quantitative RT-PCR reactions were carried out in 96-well format using ABI 7300HT instrument (Applied Biosystem) according to the instructions. Mouse 18S ribosomal RNA was used as the housekeeping gene. Data for each gene was normalized to* 18S* and presented as a ratio to the transcript of interest to* 18S*.

### 2.3. SIRT1 Activity Measurement

SIRT1 Fluorometric Drug Discovery Kit (BML-AK555, ENZO Life Science International, Inc., PA, USA) was used to measure SIRT1 activity in C2C12 myotubes, following the manufacturer instruction. In this assay, SIRT1 activity is determined by the degree of deacetylation of a standardized substrate that contains an acetylated lysine residue. This Fluor de Lys substrate is a peptide containing amino acids 379–382 of human p53 (Arg-His-Lys-Lys [Ac]) and serves as a direct target for SIRT1. SIRT1 activity is proportionally related to the degree of deacetylation of Lys-382 and the corresponding fluorescence signal changes.

Cell lysates were harvested by homogenizing cells in RIPA buffer (Sigma-Aldrich, MO, USA), which contains protease inhibitor cocktail (MP Biomedicals LLC, Solon, OH, USA) (100 : 1 v/v). After 5 seconds of ultrasonication on ice, the cell lysates were centrifuged at 12,000 ×g for 5 minutes. The supernatant was used for SIRT1 activity assessment and other experiments. According to the protocol, 5 *μ*L of cell lysate was used for the endogenous SIRT1 activity detection. Samples were incubated in a phosphate-buffered saline solution with peptide substrate (25 *μ*M) and NAD^+^ (500 *μ*M) at 37°C on a horizontal shaker for 45 minutes. The deacetylation reaction was stopped with the addition of the stop solution (2 mM nicotinamide) and developer that binds to the deacetylated lysine to form a fluorophore. Following 10 minutes of incubation at 37°C, fluorescence intensity was measured using Glomax Multi Detection System (Promega, WI, USA), with excitation and emission wavelengths of 360 nm and 450 nm, respectively. Resveratrol (100 mM) and suramin sodium (25 mM) were used as positive and negative controls, respectively. To normalize the data of SIRT1 activity, concentrations of the sample cellular protein were measured via BCA-assay (Thermo Scientific Inc, Waltham, MA, USA). Data for each sample SIRT1 activity is presented as a ratio to the protein content.

### 2.4. Cellular NAD^+^

NAD^+^ was measured in C2C12 myotubes using a colorimetric assay (Cayman Chemical Company, Ann Arbor, MI, USA) that uses an alcohol dehydrogenase reaction to reduce NAD^+^ in cell lysates to NADH and the NADH is used to reduce a tretrazolium salt substrate (WST-1) to formazan. Formazan absorbance, measured at 450 nm, is proportional to the NAD^+^ in the cell lysate.

### 2.5. Fatty Acid Oxidation

Cellular fatty acid oxidation was measured using [^3^H]-palmitate, as described in our previous studies [13]. C2C12 cells in 12-well plates were washed with 2 mL of cold PBS solution twice and incubated in 1 mL of Hank's basic salt solution containing 0.5 mg/mL BSA, 22 *μ*M-unlabeled palmitate plus 5 *μ*M [^3^H]-palmitate (32.4 mCi/*μ*m) for 2 hours. All of the reaction solutions were collected from each well, and then 200 *μ*L of 10% trichloroacetic acid and 70 *μ*L 6 N NaOH were added in the solution. Mixtures were then removed from each well and placed in corresponding poly-prep chromatography columns with 1.5 mL Dowex-1 overnight. The ^3^H_2_O that passed through the column was collected into a scintillation vial, and radioactivity was measured with a liquid scintillation counter. The protein level of each well was measured using BCA-assay (Thermo Scientific Inc., Waltham, MA, USA) and was used to normalize the palmitate oxidation data.

### 2.6. Western Blotting

Primary antibodies for total AMPK, phospho-AMPK*α* (Thr172), total ACC (Acetyl-CoA Carboxylase), and phospho-ACC (Ser79) were obtained from Cell Signaling Technology Inc. (Danvers, MA, USA). Horseradish peroxidase- (HRP-) conjugated goat anti-rabbit secondary antibody was obtained from Thermo Scientific Inc. (Waltham, MA, USA).

Following the indicated treatments, C2C12 myotubes were washed twice with ice cold PBS, and the total cell lysates were prepared using RIPA buffer plus protease/phosphatase inhibitor cocktails with 100 : 1 : 1 (v/v/v, ratio) (Sigma-Aldrich). Following a 10-minute centrifugation at 14,000 ×g, the supernatants were collected for the determination of protein content using BCA assay kit (Thermo Scientific Inc., Waltham, MA, USA) and western blotting. Equal amounts of total cell lysates (20 *μ*g) were loaded to 10% SDS-PAGE (10 cm × 10 cm, Criterion precast gel, Bio-Rad Laboratories, Hercules, CA, USA) and transferred to PVDF membrane (polyvinylidene difluoride membrane) (Bio-Rad, Hercules, CA). The membrane was incubated in 25 mL blocking buffer (1× TBS, 0.1% Tween-20 with 5% w/v nonfat dry milk) for 1 hour at room temperature. Then the membrane was incubated in TBST containing 5% dry milk with primary antibody (1 : 1000) with gentle agitation at 4°C overnight, washed three times with TBST, and incubated with TBST containing rabbit HRP-conjugated secondary antibody (1 : 5000) for 1 hour at RT. Bound antibodies were visualized by chemiluminescence (ECL Western Blotting Substrate, Thermo Scientific) and membranes were exposed to X-ray films (Phenix Research Product, Candler, NC) for protein band detection. The films were scanned using an HP Scanjet 39070 (Palo Alto, CA 94304) and stored as tagged image file format (TIFF) at 300 dpi. The protein bands were quantified by densitometry using BioRad ChemiDoc instrumentation and software of Image Lab 4.0 (Bio-Rad Laboratories).

### 2.7. Measurement of Mitochondrial Contents

Mitochondrial abundance in C2C12 myotubes was assessed by 10-N-nonyl acridine orange (NAO) dye (Life Sciences, PA, USA) according to manufacturer's instruction. After desired treatment, cells in 96-well plates were treated with 10 *μ*M NAO dye, following 2-hour incubation at 37°C in the dark. NAO is not fluorescent, but it can be oxidized into the fluorescent-NAO by oxidative species and accumulated in mitochondrial membrane. The absorbance in each well was measured at 570 nm wavelengths (Promega, WI, USA) and normalized with cellular protein level. The image of mitochondria was taken using a Nikon Eclipse Ti-E Ti-E* Fluorescence* microscope (Nikon Metrology, Inc., US) equipped with an automated stage and a 20x objective. A 3 × 3 large image scan was taken in each of 5 random fields by multichannel capture (channel 1: excitation/emission = 488/517, channel 2: excitation/emission = 550/567 nm).

### 2.8. Statistical Analysis

Data is presented as means ± standard deviation (SD). Levene's test was used to determine homogeneity of variance among groups using SPSS 21.0 statistical software (IBM, Armonk, NY) and where necessary natural log transformation was performed before analysis. Multiple comparisons were analyzed by one-way analysis of variance (ANOVA) using least significant difference when equal variance was assumed, and Games-Howell test was used when equal variance was not assumed. The independent sample* t*-test was used to compare two conditions. Differences were considered statistically significant at *P* < 0.05.

## 3. Results

### 3.1. Leucine Treatment Induced the Mitochondrial Biogenesis in C2C12 Myotubes

**Figure 1:**
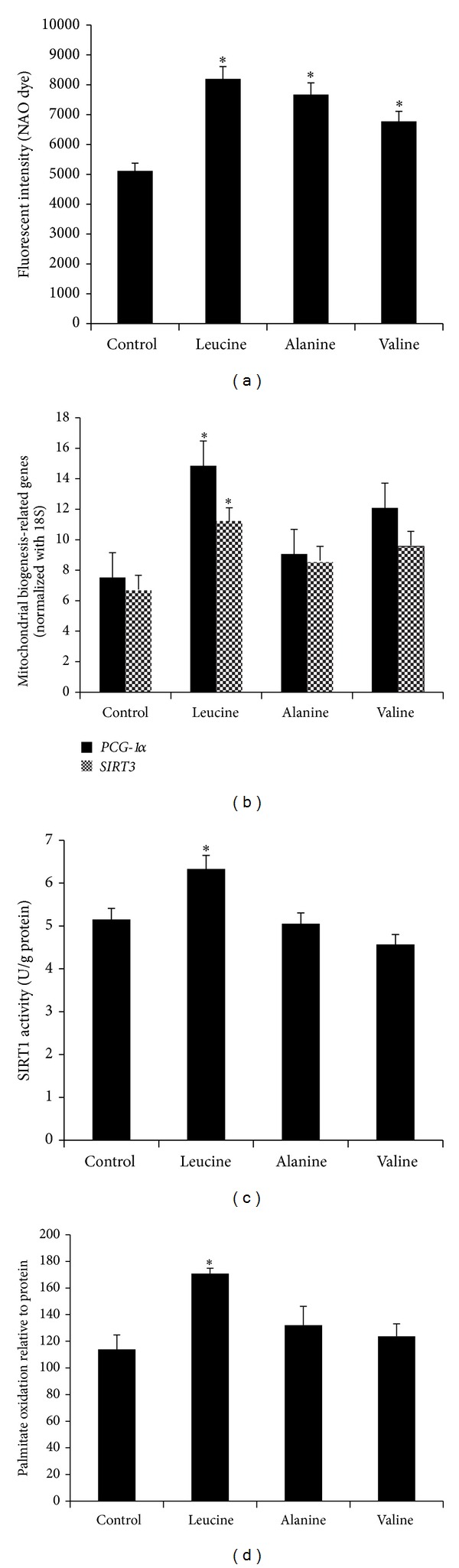
Leucine treatment induces mitochondrial biogenesis and SIRT1 enzymatic activity in C2C12 myotubes. (a) Mitochondrial content was quantitated with NAO dye (10 *μ*M) 48 hours after treatment with leucine (0.5 mM), alanine (0.5 mM), and valine (0.5 mM); (b) mRNA expression levels of* PGC-1α* and* Sirt3* with the same treatments were evaluated by quantitative RT-PCR. The relative mRNA expression was normalized to 18S and expressed as dark bars for* PGC-1α* and grey bars for* Sirt3*. (c) Cellular SIRT1 activity and (d) palmitate oxidation were measured after treatment for 48 hours. The results were normalized to cellular protein level for each sample. Data are mean ± SE (*n* = 4). *Significantly different from controls with *P* < 0.05.

Leucine significantly increased mitochondria content in C2C12s compared to alanine and valine (*P* = 0.007) (Figure 1(a)). These effects were accompanied by increases in mRNA levels of* PGC-1α* (198%, *P* = 0.01) and* SIRT3 *(167%, *P* = 0.03) (Figure 1(b)). SIRT1 activity (*P* = 0.017) and fatty acid oxidation (*P* = 0.03) in the C2C12 myotubes were significantly elevated by leucine compared to the control groups (Figures 1(c) and 1(d), resp.).

### 3.2. SIRT1 Is Required for Leucine-Induced Mitochondria Biogenesis in C2C12 Myotubes

**Figure 2:**
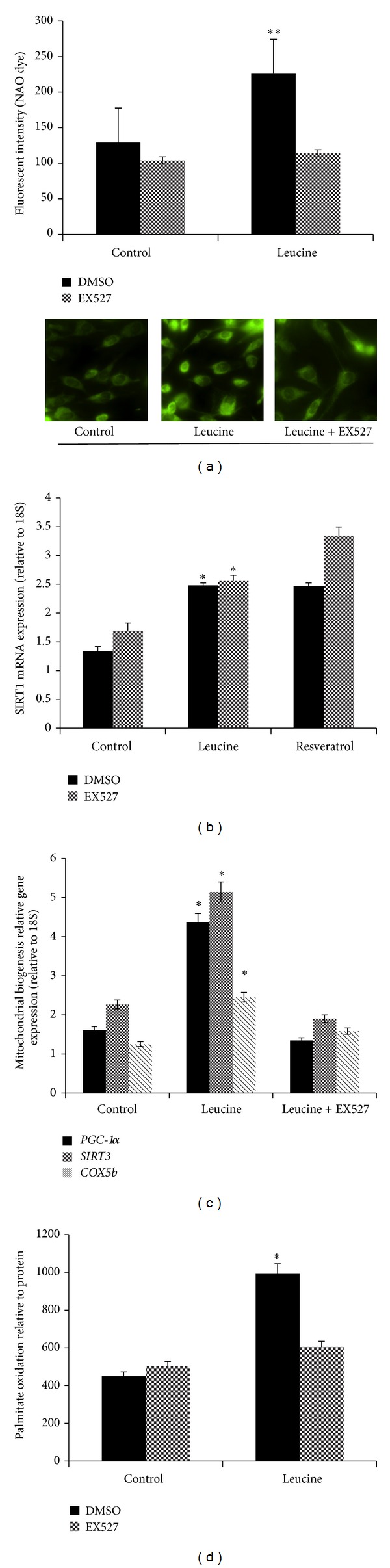
Leucine improves mitochondrial biogenesis in C2C12 myotubes in a SIRT1-dependent manner. (a) Mitochondrial content was measured using NAO (10 *μ*M) dye after 48-hour leucine (dark bars), leucine plus SIRT1 inhibitor (EX527 25 *μ*M; grey bars) for 48 hours in C2C12 myotubes. (b, c) SIRT1 activity and mitochondrial biogenesis- related genes (*PGC-1*α*, Sirt3, and COX5b*) mRNA levels were measured after the same treatments. The relative SIRT1 activity was normalized to cellular protein level, and mRNA level was normalized to housekeeper gene* 18S*. (b) Dark bars are DMSO control, grey bars are EX527. (c) Dark bars are* PGC-1α*, grey bars are* Sirt3*; striped bars are* COX5b*. (d) Palmitate oxidation level was detected after the same treatment, and the results were normalized to cellular protein for each sample. Data are mean ± SE (*n* = 4). Different letters indicate significant differences within a given variable. Dark bars are DMSO control and grey bars are EX527. *Significantly different from controls, and **significantly different from control and EX527 groups with *P* < 0.05.

We used a selective SIRT1 inhibitor (EX527) to determine the role of SIRT1 in leucine-induced mitochondrial biogenesis. Leucine increased mitochondrial biogenesis as demonstrated by significant increases in mitochondrial content (*P* = 0.001), palmitate oxidation (*P* = 0.038) and expression of mitochondrial biogenesis-related gene markers* PGC-1α*(*P* = 0.003),* SIRT3 *(*P* = 0.031), and* COX5b* (*P* = 0.015) (Figures 2(a), 2(c) and 2(d), dark panel), and these effects were markedly attenuated by EX527 administration (Figures 2(a), 2(c) and 2(d), grey panel). Comparing the relative* Sirt1* expression, leucine and resveratrol (positive control) markedly increased* Sirt1* mRNA level (*P* = 0.020); the SIRT1 inhibitor (EX527) plus leucine treatment (*P* = 0.015) revealed the same pattern (Figure 2(b)).

### 3.3. Leucine Stimulates Phosphorylation of AMPK in a SIRT1-Dependent Manner

**Figure 3:**
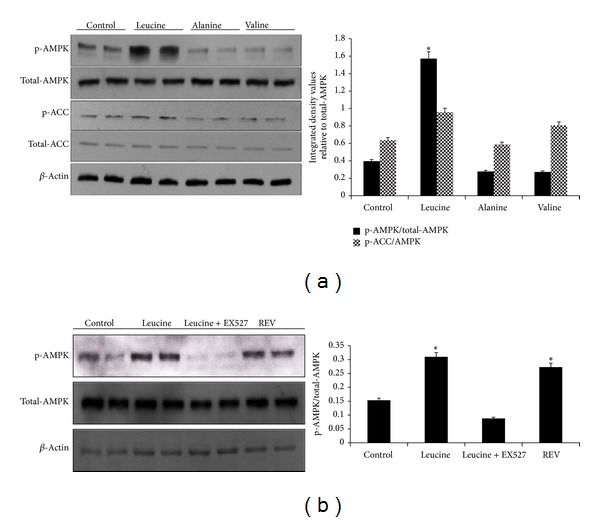
Leucine-induced phosphorylation of AMPK and ACC requires SIRT1 in C2C12 myotubes. (a) C2C12 myotubes were serum starved overnight and treated with leucine (0.5 mM), alanine (0.5 mM), valine (0.5 mM), and DMSO for 6 hours. The cell lysates were assessed by western blotting analysis with specific antibodies against phosphor-AMPK*α* (Thr 172), phosphor-ACC (Ser 79), total AMPK*α* (Thr 172), and beta-actin. Integrated density values for the p-AMPK and p-ACC were normalized to total-AMPK band density and represented as dark or gray bars. (b) C2C12 myotubes were treated with 0.2% FBS medium overnight and then treated with leucine (0.5 mM), resveratrol (100 nM), and leucine plus EX527 (25 *μ*M) for 6 hours. Whole cell lysates were prepared and detected by western blotting with specific antibodies against phosphor-AMPK*α*, AMPK*α*, and beta-actin. Integrated density value for phosphor-AMPK was normalized to total-AMPK. *Significantly different from controls with *P* < 0.05.

Six hours of leucine treatment resulted in a 3-fold increase in AMPK phosphorylation in the C2C12 myotubes, which was significantly different from baseline, valine, and alanine. Consistent with this observation, phosphorylation of ACC, a downstream target enzyme of AMPK, was also elevated by leucine compared to the controls (*P* = 0.014) (Figure 3(a)), while EX527 treatment resulted in corresponding suppression of AMPK phosphorylation (*P* = 0.012) (Figure 3(b)), indicating the necessity of SIRT1 for leucine-induced AMPK activation.

### 3.4. Leucine Stimulates SIRT1 Activity, Phosphorylation of AMPK, and Cellular NAD^+^ in a Time-Dependent Manner

**Figure 4:**
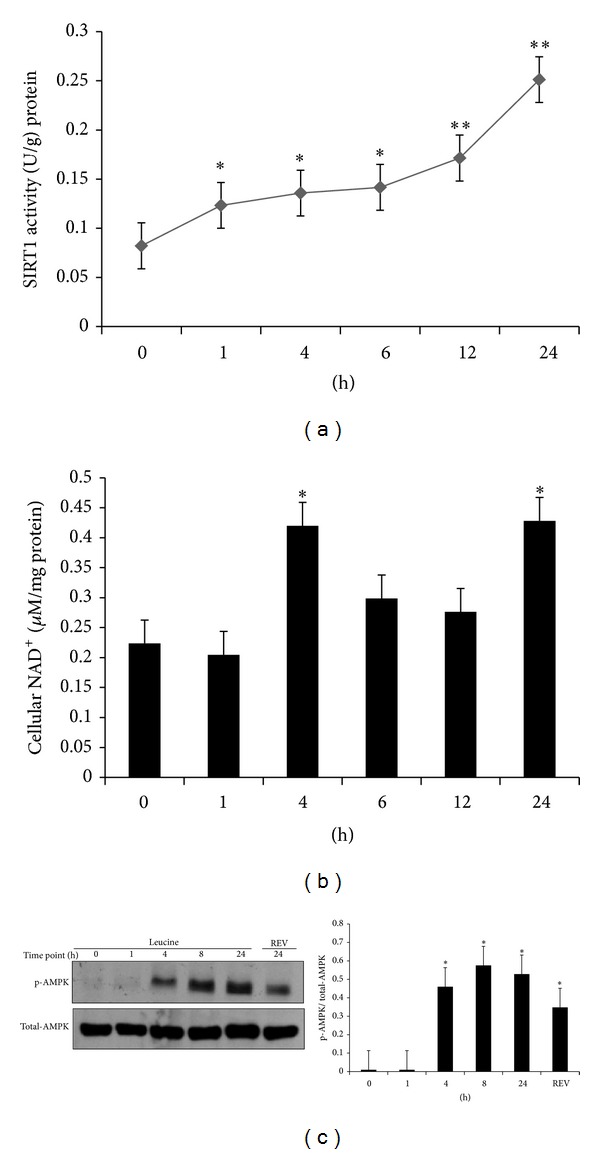
Leucine stimulates SIRT1 activity, AMPK phosphorylation, and cellular NAD^+^ in a time-dependent manner. C2C12 myotubes were serum starved overnight and treated with leucine (0.5 mM). Cell lysate was collected and analyzed for cellular SIRT1 activity, western blotting of p-AMPK and cellular NAD^+^ levels at indicated certain time points. (a) SIRT1 activity. (b) Cellular NAD^+^. Both SIRT1 activity and NAD^+^ level were normalized to cellular protein for each sample. (c) Phosphorylation level of AMPK was detected using western blotting following the same time course in C2C12 cells, with resveratrol serving as positive control. Data are mean ± SE (*n* = 3). Different letters indicate significant differences between dark or gray bars. *Significantly different from point 0, and **significantly different from time point 1.

To determine the interplay between SIRT1 and AMPK, we measured the cellular NAD^+^ level, SIRT1 activity, and phosphorylation of AMPK at time points: 0, 1, 4, 6, 12, and 24 hours by leucine treatment in C2C12 myotubes. Leucine increased SIRT1 activity at 1 (*P* = 0.028), 12 (*P* = 0.042) and 24 hours (*P* = 0.010) compared to the baseline (Figure 4(a)). However, no change was observed for the NAD^+^ content and p-AMPK level during the first 4 hours. NAD^+^ level was elevated almost twofold higher at time points 4 (*P* = 0.025) and 24 hours (*P* = 0.010) compared to baseline level and otherwise remained low (Figure 4(b)); the levels of p-AMPK were markedly increased and stayed high from 4 to 24 hours (*P* = 0.000) (Figure 4(c)).

### 3.5. Leucine-Induced Mitochondrial Biogenesis in C2C12 Myotubes Requires AMPK

**Figure 5:**
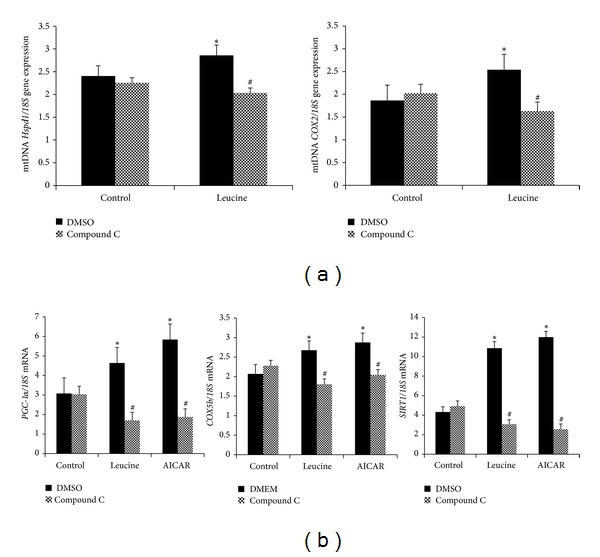
Leucine-induced mitochondrial biogenesis in C2C12 myotubes requires AMPK. (a) C2C12 myotubes were treated with leucine (0.5 mM), AICAR (20 *μ*M), and Compound C (25 *μ*M) for 24 hours. mtDNA levels of the cells were analyzed by the mitochondrial markers gene expression,* Hspd1 *and* COX2*, using real-time PCR. (b)* Sirt1* and mitochondrial biogenesis related mRNA level of* PGC-1α* and* COX5b* were evaluated also by RT-PCR after treating with leucine and Compound C for 24 hours was measured; all the mRNA levels were normalized to 18S housekeeping gene. Data are mean ± SE (*n* = 4). Dark bars are vehicle control; grey bars are Compound C. *Significantly different from controls. ^#^Significant Compound C effects.

We next examined whether AMPK also mediates leucine's impacts on mitochondrial biogenesis in C2C12 myotubes. As shown in Figure 5, leucine treatment markedly increased the mitochondrial component genes expression (Figure 5(a), dark columns),* Hspd1* (*P* = 0.003) and* COX2* (*P* = 0.003). Similar effects were found for genes encoding mitochondrial biogenesis regulatory proteins, [*PGC-1α* (*P* = 0.001),* Sirt1* (*P* = 0.022)] and component proteins [*Cox5b *(*P* = 0.04)], (Figure 5(b), dark columns), while Compound C treatment markedly impaired all these inductions (Figure 5, grey panels).

## 4. Discussion

These data indicate that leucine stimulates significant muscular metabolic changes, including SIRT1 activation, AMPK phosphorylation, and mitochondrial biogenesis in C2C12 myotubes. These changes may contribute to leucine's beneficial effects on energy metabolism and insulin sensitivity in both animal and human models [17, 19, 27, 28].

A previous clinical trial has shown that high dairy intake (rich in BCAAs) induces significant suppression of reactive oxygen species (ROS) and inflammatory stress, indicated by decreased plasma tumor necrosis factor alpha (TNF-*α*), interleukin 6 (IL-6), and monocyte chemoattractant protein-1 (MCP-1) levels [19]. Doubling leucine intake in mice has been found to reverse multiple HFD-induced metabolic abnormalities, including glucose intolerance, hepatic steatosis, and inflammation [11]. These effects are accompanied by corresponding increases in mitochondrial oxidative capacity and mitochondrial content. Since mitochondrial dysfunction and mitochondrial content loss are directly linked to the development of metabolic disorders [1, 29], increased mitochondrial biogenesis appears to rescue part of these obesity-related abnormalities [30].

Consistent with our previous studies [13], here we show that 0.5 mM leucine treatment, which is comparable to the plasma leucine concentration achieved by a leucine-rich diet [31], can markedly increase mitochondrial content, mitochondrial biogenesis-related gene expression, and fatty acid oxidation in C2C12 myotubes, compared to valine and alanine.

The data herein demonstrate that the improvement of fatty acid oxidation and mitochondrial content by leucine is accompanied by increased SIRT1 activity in C2C12 cells. SIRT1 has been demonstrated to play significant roles in leucine's effects on energy metabolism. In Macotela's study [11], leucine restores HFD-reduced hepatic NAD^+^ and SIRT1 expression back to normal levels. Similarly, Li et al. demonstrate that leucine increases SIRT1 expression and decreases acetylation level of PGC-1*α*, resulting in attenuation of HFD-induced mitochondrial dysfunction, insulin resistance, and obesity in mice [32]. Furthermore, Sun and Zemel found that leucine induces mitochondrial biogenesis in muscle cells by stimulating the expression of PGC-1*α* and NRF-1 via a SIRT1-dependent pathway [26]. These findings, along with the observations reported here, are in agreement with our recent work that leucine could activate SIRT1 enzyme through allosteric interaction in adipocytes and myotubes [25].

To establish whether or not SIRT1 is required for leucine-induced mitochondrial biogenesis, EX527, a selective SIRT1 enzyme inhibitor, was used to treat the cells in combination with leucine. EX527 significantly attenuated leucine-induced mitochondrial content, mitochondrial biogenesis-related genes expression, and fatty acid oxidation in C2C12 myotubes. The observations reported here are consistent with Price's work,in which SIRT1 knockout completely blocked resveratrol-induced mitochondrial biogenesis and *β*-oxidation in skeletal muscle [33], further supporting the essential roles of SIRT1. However, the leucine-induced* Sirt1* gene expression was not affected by EX527, possibly due to the unique inhibition mechanism of EX527 on SIRT1 catalytic activity [34].

We also found that AMPK phosphorylation, which is elevated in response to metabolic stress [35], was also increased by leucine in C2C12 myotubes. This change might help to explain the increased fatty acid oxidation in the cells. Similarly, in mice, leucine supplementation has been reported to activate AMPK synergistically with resveratrol and metformin, resulting in increased insulin sensitivity and glucose tolerance [22]. On the other hand, Compound C, an inhibitor of AMPK, markedly blocked leucine's effects on mitochondrial biogenesis, indicating that like SIRT1 elevated mitochondrial biogenesis and fatty acid oxidation by leucine requires AMPK in C2C12 myotubes.

Notably, we found that leucine-induced AMPK phosphorylation was markedly blocked by EX527, suggesting that AMPK might serve as a downstream target of SIRT1. In support of this concept, Price et al. reported that SIRT1 activation is required for AMPK phosphorylation and improvement of mitochondrial function via deacetylation and activation of LKB1, a primary upstream kinase of AMPK [33, 36], while Park et al. found that resveratrol activates SIRT1 via an indirect pathway involving calmodulin-dependent protein kinase kinase *β* (Camkk*β*) and AMPK activation [36]. Currently available evidences suggest that AMPK and SIRT1 display mutual interactions with each other; AMPK could activate SIRT1 by increasing cellular NAD^+^ level via promoting expression of nicotinamide phosphoribosyltransferase (Nampt), a rate-limiting enzyme in NAD^+^ biosynthesis; however, SIRT1 can also directly deacetylate and activate LKB1, resulting in the activation and phosphorylation of AMPK [37].

Our time-course data suggest that SIRT1 may be the initial target of leucine. SIRT1 activity was increased within the first hour of leucine treatment, while cellular NAD^+^ and p-AMPK levels remained unchanged. Considering that the increased* Sirt1 *mRNA and SIRT1 activity level occurred at some time after the leucine treatment for 24 hours, it is possible that SIRT1 activity is elevated by leucine first, and then activation of AMPK is a subsequent event, which may be responsible for the further SIRT1 activation at the later time points.

Our data may also reflect dose-dependent effects of leucine treatment. For example, high-dose leucine infusion and supplementation have been shown to induce insulin resistance and glucose intolerance in both human and animal models [38, 39], possibly via activation of mammalian target of rapamycin- (mTOR-) insulin receptor substrate 1 (IRS-1) signaling pathways [40]. In contrast, modest increases in leucine intake, sufficient to induce plasma leucine elevations to ~0.5 mM, significantly reduced obesity-related oxidative and inflammatory stress, resulting in improvement of insulin sensitivity in humans [19]. Similarly, Vaughan et al. found that leucine in the 0.1–0.5 mM range induces a dose-dependent increases of PGC-1*α* expression, leading to significant elevated mitochondrial density and oxidative capacity in skeletal muscle cells [17]. Consistent with these evidences, we found comparable levels of leucine promoted mitochondrial biogenesis and fatty acid oxidation in C2C12 myotubes.

There are several limitations to this study. One of them is the use of the Fleur de Lys assay to measure SIRT1 activity. Studies have challenged the validity of the assay, as some of them have found that sirtuin-activating compounds (STACs) only increased SIRT1 activity by using fluorophore-tagged substrates but not the matching nontagged peptides, which also might explain why the activation can be found exclusively* in vitro *but not* in vivo* [41, 42]. According to Gertz et al., the fluorophore can act synergistically with STACs to promote binding between substrates and SIRT1 enzyme [43]. Furthermore, evidence suggests that resveratrol-induced SIRT1 activation is actually mediated through an indirect signaling pathway involved in cAMP phosphodiesterases (PDE) and AMPK* in vivo* [36]. However, Hubbard et al. recently provided more evidences to support the allosteric binding and activation theory between STACs and SIRT1. They found that specific hydrophobic motifs in SIRT1 substrates and a single amino acid (Glu^230^) in SIRT1 enzyme mediate the structure change during the deacetylation [44]. As a highly hydrophobic amino acid, leucine might directly activate SIRT1 through conformation change. Indeed, recent evidence demonstrates that leucine exerts direct effects on SIRT1 kinetics by decreasing 50% km for NAD^+^ and substrates. With the presence of leucine and HMB, lower concentration of resveratrol is required for the activation of SIRT1 [45]. Therefore, further experiments using fluorophore-free substrates to measure the SIRT1 activity are needed to elucidate the exact pathways of leucine-activated SIRT1. A second limitation is lack of data assessing the cellular acetylation status of LKB1 and PGC-1*α*, as well as Nampt phosphorylation and expression.

**Figure 6:**
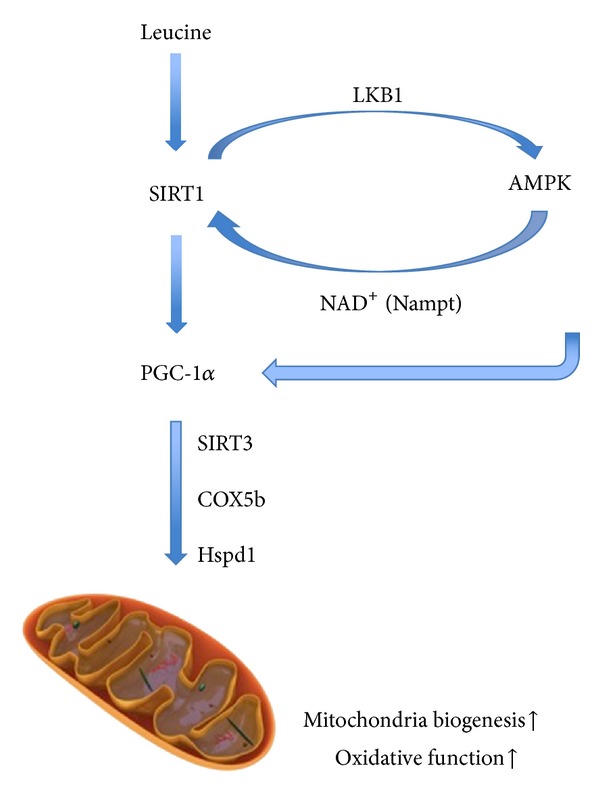
Proposed mechanism of leucine-induced mitochondrial biogenesis. In C2C12 myotubes, leucine treatment leads to activation of SIRT1. SIRT1 then deacetylates and activates LKB1, which subsequently induces AMPK phosphorylation and activation. In turn, activated AMPK could promote SIRT1 activation via intracellular NAD^+^ level by changing expression and activity of Nampt. Activated AMPK and SIRT1 further activate PGC-1*α* via phosphorylation and deacetylation, resulting in elevated mitochondrial biogenesis and oxidative function.

In summary, with the present work, we demonstrate that leucine improves mitochondrial biogenesis and fatty acid oxidation in C2C12 myotubes through SIRT1 and AMPK-dependent pathway, with secondary activation of AMPK mediated by SIRT1 (Figure 6).
